# Arrangements in Edinburgh and Dundee: Dundee—Edinburgh: Arrival of Navy Men

**Published:** 1914-09-12

**Authors:** 


### Arrangements in Dundee and Edinburgh.

DUNDEE.

The Dundee Branch of the British Red Cross Society is putting itself in readiness under the directorship of Colonel Wm. Gordon Thomson, the hon. secretary, who is a Red Cross commissioner for the Central Eastern District, Scotland.

The directors of the Royal Infirmary have guaranteed 155 beds to the Admiralty, and as many more for the treatment of wounded soldiers as can possibly be spared. The majority of the beds are ready.

The Parish Council has placed a portion of the Eastern Poorhouse Hospital, capable of accommodating 130, at the disposal of the Branch. Everything is in readiness for immediate service, and a fully equipped operating theatre has also been handed over to the Society.

It has been decided to reserve the accommodation at King's Cross Hospital for the treatment of any sailors or soldiers who may have contracted an infectious disease. In this connection Dr. Templeman, the city medical officer of health, has promised to give all the assistance and attention he possibly can. The fitting-up of the Caird Rest as a hospital for fifty beds is almost complete.

Mr. R. B. Don, a prominent Dundee manufacturer, has given the Branch the use of his Broughty Ferry house, The Lodge, capable of accommodating forty. This small and well-equipped hospital is ready. Among other proposals, Messrs. Cox Brothers, the proprietors of the well-known Camperdown Jute Works, Lochee, have offered to fit out their schools, and a hospital in miniature for fifty patients, complete in every detail, is now practically ready.

The War Road Gymnasium and the Seamen's Reading Room, Dock Street, have been fitted out as dressing stations, where wounds of a less serious nature will be dressed.

The various improvised hospitals will be staffed by the members of the four Voluntary Aid Detachments of the Scottish Territorial Red Cross Brigade, and many of the local medical men have offered their services.

When the preparations of the Red Cross Society are completed, the command will be taken over by Colonel Wyville Thomson, who is senior medical officer in command of the Tay Defences.

EDINBURGH: ARRIVAL OF NAVY MEN.

Eleven naval patients have been admitted to the Royal Infirmary, Edinburgh, these being a proportion of the cases of sickness or accident brought into the Firth of Forth by one of the hospital ships from the flotilla on duty in the North Sea. Twelve military patients have been dealt with, these being likewise cases of sickness or accident, but no war casualties have yet been sent in.

